# Narratives of Transgender People Detained in Prison: The Role Played by the Utterances “Not” (as a Feeling of Hetero- and Auto-rejection) and “Exist” (as a Feeling of Hetero- and Auto-acceptance) for the Construction of a Discursive Self. A Suggestion of Goals and Strategies for Psychological Counseling

**DOI:** 10.3389/fpsyg.2017.02367

**Published:** 2018-01-17

**Authors:** Alexander Hochdorn, Vicente P. Faleiros, Paolo Valerio, Roberto Vitelli

**Affiliations:** ^1^Post-graduation Program in Psychology, Catholic University of Brasília, Brasília, Brazil; ^2^Department of Neuroscience and Reproductive and Dental Sciences, University of Naples Federico II, Napoli, Italy

**Keywords:** transgender women, prison, psychological counseling, clinical psychology, healthcare policies, violence prevention, text-mining analysis

## Abstract

**Purpose:** Understanding how transgender people, who committed criminal offenses and are detained in prison, produce a narrative representation of self within different prison contexts. More specifically, this study has been based on two sub-aims: On a paradigmatic level, it has been aimed at critically investigating how the discursive positioning among the Self and the Other might promote the internalization of positive and/or negative attitudes toward the self. On a pragmatic level, it intends to offer some suggestions for goals and strategies of psychological counseling with these inmates inside such highly institutionalized contexts.

**Method and Materials:** In total, 23 in-depth interviews were conducted with transgender women detained in either female or male prison contexts in Italy and Brazil. The lexical, semantic, and semiotic structure of the transcribed interviews has been investigated by adopting the quali-quantitative software *Iramuteq* for performing statistical text-mining analysis. Frequency, correspondences, and distribution of the most representative utterances across the corpus of data have been accessed and critically analyzed.

**Results:** The findings showed that transgender inmates in Brazil made repeated use of the adverb “not,” while the verb “exist” became the most representative word for the Italian sample. In Brazil, indeed, transgender women assumed masculine-driven behavior due to a common imprisonment with cis-gender men. On the contrary, transgender women in Italy are detained in protected sections, where they are allowed to wear female clothing and continue hormonal treatments. Surprisingly, transgender inmates in Italy suffered more violence in a female sector when compared to exclusively male jails.

**Conclusions:** Transgender people represent a challenge for prison administration because it is not clear in which penitentiary context they should be detained. They should receive special attentions in order to face their special needs, which are radically different when compared to other typologies of inmates. Within penitentiary contexts, psychological counseling with transgender women should pay a special attention to the several psycho-social dimensions of this existential condition. In particular, psychological counselors should consider its inner complex articulation within different social, cultural and normative contexts.

## Introduction

Transgenderism refers to people who have a gender identity not fully aligned with the gender assigned at birth (American Psychological Association, 2015). The origin of this condition is unknown still today. Different hypotheses have been proposed regarding the possible influence of genetic and biological/hormonal factors eventually affecting subjects as early as during the first intrauterine development, as well as of specific early rearing experiences (Zucker et al., 2016). In any case, it is clear that also in this case, pre-verbal inner experiences are afterwards only discursively signified within that horizon of meaning, opened up by social discourses, i.e., by a culturally and therefore historically determined backgrounds. McAdams (2001) outlined that “people living in modern societies provide their lives with unity and purpose by constructing internalized and evolving narratives of the self” (p. 100). In recent decades, a proliferation of psychological research on narrative identity has provided a strong empirical basis for the construction of a discursive identity (Staudinger, 2001; Bauer et al., 2005; McLean et al., 2007; McLean, 2008; McLean and Fournier, 2008; Adler, 2012; McAdams and McLean, 2013). Indeed, it is only by means of linguistic universes that subjects frame their experience making it meaningful to themselves and the others, and finally interpret their own identities (Berger and Luckmann, 1966). Language alone provides the framework according to which we define the rules and restrictions that form the set of beliefs, preferences, attitudes and behaviors of the social actors; in other words, exactly what, when it comes to sexual gender, scientific and non-scientific literature indicates as gender identity. Referring to Binswangerian theories it has been outlined, that in many cases the transgender's world-project, articulated around the signifier of “Feminity/Masculinity,” structures their whole field of life-experience (Vitelli, 2015). What is more commonly at issue in such cases, is a model of femininity/masculinity borrowed from a general type of femininity/masculinity that is already crystallized in public life, and, consequently, common sense. At the same time, it must be considered how “transphobic instances, widespread in Western countries (as well as in many other parts of the world) are introjected” by subjects. Thus, different contextual situations, sometimes also within the same context, shape different representations of the self, demonstrating that identity is circumscribed by situated practices of interaction and positioning.

In a paper published by Jenness and Fenstermaker (2014) about the detainment conditions of women-affirmed transgender people in the Californian penitentiary system, the authors commented on an extract out of the interviews they gathered, that “whether transgender women actually fight or not—and the data suggest they do—is not the point; the point is the fact that they render fighting sensible through a gendered lens” (p. 22). Such a statement perfectly reflects a silent and, at the same time, difficult challenge to be faced either by transgender people themselves, detained in prison, as by the staff that interacts daily in these institutions (Hochdorn et al., 2015). Transgender people, indeed, have to position themselves constantly according to a heteronormative socio-cultural hegemony, conceiving just two gender antipodes in an isomorphic relation with the biological sexes (West and Zimmerman, 2009; Vitelli, 2015). Such a condition often promotes extremely harsh feelings like inappropriateness, exclusion, isolation, and marginalization at all levels of the social structure, from private to public contexts (Schilt and Westbrook, 2009; Connell, 2010; Hochdorn et al., 2016).

Certainly, such negative representations of transgender people in culture and society are exacerbated in highly institutionalized environments like prison (Alexander and Meshelemiah, 2010; Sexton et al., 2010; Brown, 2014; Jenness, 2014). The penitentiary context itself was conceived to exclude, punish, and change a so-called deviant person's behavior (Foucault, 1995). As transgender people trespass both an institutional order (the crime they committed) and a symbolic order (their undoing gender; Hochdorn et al., 2016), they already suffer a double punishment for being either a transgressor or a transgender (Sexton et al., 2010). Their detention period, consequently, becomes particularly difficult, especially if they are co-located together with cis-gender prisoners:

From the point of view of those charged with managing prisons, transgender prisoners are often thought to be the source of in-prison disorder and attendant management problems precisely because they do not conform to the dictates of an extremely heteronormative and masculinist environment (Jenness, 2014: 10).

Self-harm, auto-mutilation, and auto-castration become in some cases the last desperate attempt for denouncing the lack of recognition for their own personal right of intimate and social citizenship (Brown, 2010).

In addition, a third variable problematizes even more the marginal condition of transgender people in jail: being black. As already pointed out by some scholars (Reisner et al., 2014; Stotzer, 2014), being incarcerated, transgender, and black reinforces a situation of further rejection. The struggle emerging from such an intersectional stigma makes black transgender women fight for recognition for both race and gender. To the best of our knowledge, however, only a few empirical studies (Hochdorn et al., 2015, 2016) have critically analyzed the complex situation of black transgender inmates directly in prison, even if some research clearly showed evidence of the strict association between skin color and detention (Graff, 2015; Constantino et al., 2016).

Interestingly, most of studies conducted on black transgender people in jail have focused on the correlation between HIV and unprotected sexual behavior. This research, indeed, has highlighted that people of black skin color represent among the highest percentages of socially marginalized categories, like prisoners or HIV-affected people, when compared to other racial groups (Stephens et al., 1999, 2003; Phillips and Patsdaughter, 2010; Brewer et al., 2014).

Such data sheds light on the urgent need to consider the intersectional complexity between sex, gender, race, and class in order to understand the way transgender people are represented throughout the gendered lens of a dominant and heteronormative binary that is historically anchored within the normative and symbolic organization of most societies worldwide and survives even today (Bourdieu, 2001).

Understanding the modalities of interaction from micro (cognitive functions and emotions) to macro (culture and society), allows on a pragmatic level, moreover, to identify coping strategies to be implemented in healthcare policies and clinical intervention programs, specifically addressed to transgender people in jail. As shown by several studies focused on this topic (Brown and McDuffie, 2009; Chianura et al., 2010; Brown, 2014), transgender people in prison request special needs, whether compared to other typologies of prisoners. Transgender inmates, indeed, are generally more exposed to processes of violence and exclusion, and such a harsh existential condition exacerbates even more within highly institutionalized contexts, such as prisons.

In order to prevent self-harm, loose of identity, and psychotic diseases due to their detention period, specific multi-professional intervention strategies, among clinical psychologists, psychiatrist, nurses, endocrinologists, general practitioners and educators, must be promoted in prison in order to improve either the detention condition of transgender people themselves or the professional practices of those prison-workers, who daily interact with such inmates.

## Literature review

Most of the contributions that have focused on transgender people in prison have been conducted in the USA (Sultan, 2003; Brown and McDuffie, 2009; Alexander and Meshelemiah, 2010; von Dresner et al., 2013).

These publications, furthermore, mainly belong either to healthcare or social sciences, adopting mostly clinical (Samons, 2001; Brown and McDuffie, 2009; Alexander and Meshelemiah, 2010), behavioral (Petersen et al., 1996; Stephens et al., 2003), or systemic (Chianura et al., 2010) approaches for the first, and post-structuralist ones (Jenness, 2010, 2014; Jenness and Fenstermaker, 2014) for the second scientific area. Other studies, moreover, have principally been focused on endocrinological (Valenta et al., 1992) and epidemiological—basically HIV (Stephens et al., 1999, 2003; Phillips and Patsdaughter, 2010; Brewer et al., 2014)—factors associated with conditions of transgender people's detainment.

Studies about imprisonment, considering transgendering paths as an issue of clinical interest (Alexander and Meshelemiah, 2010), are mostly focused on the treatment that transgender people should receive in prison in order to complete their physical transition. Such approaches are still anchored in a quite dualistic perspective, considering gender as superimposable to sex and transgender people, who, therefore, should define their identity throughout surgical and hormone-based treatments to transform their phenotypical sexual characteristics into the opposite ones. Such an etiologic view, based on the idea of an internal personality trait felt as strongly dissonant according a “biological mistake,” which should be solved and corrected through clinical (medical and psychotherapeutic) treatments, is common to most studies in this area:

Clinical observations suggest that male-to-female (MTF) transgendered individuals often use contragender-negative reinforcement to conceal their transgender feelings (…) or may exhibit hypermasculine behaviors that serve as a facade to conceal his internal feminine feelings. He may make commitments in his male gender role that are difficult to alter at a later time in his life, when making changes toward a female gender role may appear more desirable (Samons, 2001: 143).

This quotation clearly shows the way transgender people are imprisoned, rather than in jail, within a sexualized, deterministic ontology, making it very difficult for these people to claim their right to intimate citizenship. The continued references to “male to female” transition, “treatment,” “gender identity disorder,” and above all, the use of terms such as “he” and “male toward female gender roles,” along with improper semantics like “his feminine feelings,” even considered as “more desirable” (Samons, 2001: 143), are going to emphasize a stereotyped, heteronormative

system of meanings and symbols—and the rules, privileges, and punishments pertaining to their use—for power and sexuality: masculinity and femininity, strength and vulnerability, action and passivity, dominance and weakness. One can see in it the outlines of something that links misogyny, homophobia, transphobia, and the restricted way we raise our youth (Wilchins, 2002: 25-26).

Such a vicious circle among common sense and scientific production promotes a widespread representation of transgender people linked to diversity, difference, and deviance, where “social interactions can reflect and reiterate the gender inequality characteristic of society more generally” (Fenstermaker et al., 2002: 28).

## Background

In the United States, and also in Italy and Brazil, prison is based on such a binary system, conceiving only two main circuits with regard to gender identity: the male and the female one (Chianura et al., 2010). Both the Italian or the Brazilian penitentiary system, moreover, predispose several so-called protected sections within the ordinary system, where people, classified with regard to a particular affirmation of identity, typology of crime or life condition, are detained. These sections in Italy normally correspond to the following categories of inmates: people who committed crimes of a political entity (“Brigate rosse”; Red Brigades), organized crimes (Mafia), drug-addicts, sex-offenders (rape and pedophilia), people with a diagnosis of psychiatric diseases, mothers with children up to 3 years old, homosexuals, and finally people whose gender identity falls somewhere in between or outside the sexual dualism. For them, so-called protected sectors have been instituted in eight prisons, according to the latest available survey in 2009, namely Naples, Rome, Florence, Belluno, Rimini, Alba, Milan, and Bollate for 78 inmates in total, mainly of Brazilian nationality (Chianura et al., 2010). In all these penitentiary structures the sections for transgender inmates are inserted inside the male section, except in Florence, where the sector is co-located within the female part of the prison (Hochdorn and Cottone, 2012; Hochdorn et al., 2015).

The Brazilian penitentiary system also predisposes separated sectors for inmates identified with so-called special needs. As in Italy, these sectors are designated for criminals who could present a risk for themselves (child-abusers, police officers/criminal informants) or for other inmates (leaders of criminal organizations, drug-dealers). Also, mothers with babies up to 6 months receive special attention and are kept apart from the remaining female population. However, only a few prisons in Brazil predispose special sections for homosexual or transgender prisoners. Their detention exclusively depends on the discretional decision of each prison's directors, because no national legislation is available. In the second federal prison of Brasília (PDF II), belonging to the *Papuda* penitentiary complex, the seven transwomen interviewed for the current study are detained inside a unique cell along with bi- and homosexual men, for a total of 22 inmates.

Until today, indeed, it has not been clear how to solve a situation where symbolic constraints do not manage a gender claim, in contrast to the heteronormative hegemony of prisons' organizational logic. Even regarding the Italian context, where special attention to transgender people's detainment has been paid since the 1980s, little has been done (Chianura et al., 2010; Hochdorn et al., 2016).

## Objectives

Due to the lack of scientific knowledge, along with the difficult challenges that inmates must face every day in these highly institutionalized contexts, the current research intends to offer an integrated perspective about such a segregated, ignored, and stigmatized reality, considering the intersectionality among gender, race, and class. Such an interdependency highlights the urgent need to turn this silent and complex reality visible, promoting at the same time relevant implications for healthcare and social policies on one hand and an important contribution to gender and cultural studies on the other hand.

Accordingly, the objective of the current research consists of the understanding of how transgender people are doing, undoing, or redoing gender in Brazilian and Italian jails, by investigating whether gender in such contexts results being reified by specific rhetorical practices while talking about their life in transition, not only between gender (man and woman) but also between cultures (Italy and Brazil) and races (black and white).

Due to the observed lack of studies on transgender people in jail, moreover, this study aims to show that different situations, within the same context, shape different representations of the self, demonstrating that identity is circumscribed by situated practices of interaction and positioning. The wired interrelation among context (prison), situated positioning (everyday interactions in jail), and norms (penitentiary system) has, therefore, been analyzed in order to understand if (trans)gender identities, though being reified by a dominant sexual binary, could be considered in an intersectional perspective, as strictly associated with specific ethnic groups and social classes.

Finally, the current research presents the first direct and participant observation of a reality of extreme psychological, sociological, cultural as well as normative relevance and of great topicality, along with the intersectional importance of gender and social policies—that of transgender people detained in different penitentiary contexts—to whom no integrated and unitary workspace has been devoted until today in Italy and Brazil.

## Methods and materials

### Sample characteristics

Drawing on the theoretical basis previously expressed with regard to the on-going development of personal identity and, specifically, on the idea of a narrative construction of personal identity, a quali-quantitative approach of text-mining analysis was adopted. It is noteworthy that different qualitative researches have been already carried out in recent years because they have been proved to be effective in critically analyzing discursively reified patterns of identity such as the gender transition paths investigated in the current research (Vitelli, 2015; Cipolletta et al., 2017). In total, 23 in-depth interviews were conducted from 2012 to 2016 with transgender women detained in either Italian or Brazilian prisons.

1. Brazil: Seven interviews were conducted in the second penitentiary unit of the federal male prison “A Papuda” in Brasília, Brazil. This number corresponds to the entire transgender population that has been detained during the data collection.

For a detailed description of each interviewee's information, see Table 1.

**Table 1:** Sample characteristics of all transgender inmates detained in the Brazilian prison.

**Inmates**	**Crime**	**Length of detention**	**Work in prison**	**Times of detention**	**Origin**	**Age (years)**
**FEDERAL MALE PRISON “A PAPUDA” OF BRASÍLIA**						
1. Inmate	Theft	6–7 years	None	1st	Brasília	24
2. Inmate	Drug dealing	6 years	None	1st	Maranhão	29
3. Inmate	Drug dealing & theft	4 years	None	1st	Belo Horizonte	35
4. Inmate	Drug dealing	5 years	None	1st	Maranhão	33
5. Inmate	Drug dealing	5 years	None	1st	Goiânia	31
6. Inmate	Alteration of rubbed cars' registration number	1 year	None	1st	Maranhão	19
7. Inmate	Sex-work & drug dealing	4 years	Cleaning	2nd	Belém	36

2. Italy: A total of 16 interviews were conducted in three Italian prisons:
  - The male prison “Villa Baldenich” in Belluno, where all four transgender women, detained during data collection, were interviewed. Three were from Brazil and one from Italy.
  - The male prison “Poggioreale” in Naples, where all seven transgender women, detained during data collection, were interviewed. One was from Brazil, one from Algeria, while all others were from Italy.
  - The new penitentiary complex of Florence-Sollicciano, where five Brazilian inmates out of 18, detained at that moment in a separate section of the prison's female ward, were interviewed.

For a detailed description of each interviewee's information, see Table 2.

**Table 2:** Sample characteristics of all transgender inmates detained in the Italian prisons.

**Inmates**	**Crime**	**Length of detention**	**Activity in prison**	**Times of detention**	**Origin**	**Age (years)**
**MALE PRISON OF BELLUNO-BALDENICH**						
1. Inmate	Evasion	2 years	Student	2nd	Manaus (Brazil)	25
2. Inmate	Assault	3 years	Manufacturing	1st	São Paulo (Brazil)	40
3. Inmate	Assault	4 years	Manufacturing	1st	Rio de Janeiro (Brazil)	36
4. Inmate	Multiple crimes	1–2 year(s)	Cleaning	4th	Naples (Italy)	50
**MALE PRISON OF NAPLES-POGGIOREALE**						
1. Inmate	Drug dealing	1–2 year(s)	Cleaning	1st	Rio de Janeiro	24
2. Inmate	Theft	4–5 years	Cleaning	2nd (at least)	Algeria	47
3. Inmate	Sex offender	3 years	Manufacturing	1st	Salerno (Italy)	32
4. Inmate	Drug dealing	1 year	Cleaning	1st	Naples	51
5. Inmate	Multiple crimes	3 years	None	14th	Cosenza (Italy)	35
6. Inmate	Pluri-homicide & Organized crime	Life sentence	None	3rd	Naples	50
7. Inmate	Drug dealing	1–2 year(s)	Cleaning	1st	Naples	22
**FEMALE WARD OF THE PENITENTIARY COMPLEX OF FLORENCE-SOLLICCIANO**						
1. Inmate	Sex-work exploitation & drug dealing	5 years & 6 months	Cleaning	2nd (at least)	Minas Gerais	31
2. Inmate	Sex-work exploitation	1 year & 8 months	Student	3rd (at least)	São Paulo	35
3. Inmate	Extortion & drug-dealing	6 years	Cleaning	1st	Minas Gerais	40
4. Inmate	Theft & extortion	Awaiting trial	Student	1st	São Paulo	25
5. Inmate	Sex-work exploitation	4 years	Clerk in the jail's store	2nd (at least)	São Paulo	26

#### Declaration of ethical conformity

This study was carried out in accordance with the recommendations of 'name of guidelines, name of committee' with written informed consent from all subjects. All subjects gave written informed consent in accordance with the Declaration of Helsinki. In particular, all participants took part voluntarily in the present study, and informed consent forms explaining the detailed goals of the research were distributed to all subjects before being interviewed. Sampling, methods, and analytical procedures were in full compliance with the ethical guidelines established by the National Board of Italian Psychologists (Ordine Nazionale Psicologi, 1989) and the Brazilian Federal Board of Psychology (Conselho Federal de Psicologia, 2005). For all interviews that were conducted in the Italian prisons, the appropriate permissions were obtained from the local Departments of Prison Administration of Veneto, Campania, and Tuscany, while for those conducted in the Brazilian jail, permission was obtained from the National Penitentiary Department. The current study was approved by the committee of ethics of the University of Naples, “Federico II” (protocol number: 6920).

### The prison contexts

The influence of context on discursive production, along with its implicit, symbolic meanings, becomes extremely important in order to investigate all realities concerning interaction among people, culture, and society. For defining research goals and data, direct observation of the prisons of Brasília, Belluno, Naples, and Florence has, therefore, been performed, previous to the interviews with the main agents of the study. Such an analysis should provide important information with regard to different frames of interaction, namely the historical, social, urban, and normative structure, in which each of these institutions is embedded (Zucchermaglio, 2004).

#### Brasília-Papuda

The federal male prison “A Papuda” of Brazil's capital Brasília is located in a satellite-town, São Sebastão, inside Brasília's federal district. This enormous prison complex is split into two sub-units [1st and 2nd penitentiary unit (PDF I and PDF II, respectively)] and hosts a total of nearly 6,500 inmates (Da Silva Magalhães, 2017). The data was collected between August and September 2015 in Block G of the PDF II, where all seven transgender people were located in the same cell together with other 14 homosexual cis-gender men. According to the Brazilian legislation, indeed, inmates must be detained in the prison wards that correspond with their civil sex. As transgender women who have not already completed their sex-reassignment surgery are still classified as men, they are located in ordinary sections within male jails. Furthermore, in Brazil, prison uniforms for inmates are still used and as for all other male prisoners, transgender women must wear male clothes. They also must cut their hair and interrupt eventual hormonal therapies. They are allowed even less to use female make-up. Only recently, from the prison director's personal decision, they have been located in a unique GBT cell; only in this place can they now wear female clothes.

#### Belluno-Baldenich

This quite particular jail (“Casa circondariale”) was instituted in 1933 and is classified as a low-access male prison (“carcere a bassa soglia”). The overall number of inmates, indeed, was just 93 people during data collection, out of whom four have been identified as transgender women and have, therefore, been detained in a separate wing of the institution (Ministero della Giustizia, 2017). All transgender inmates have individual cells with a separate bathroom (complete with shower, bidet, and hot water), television, desk with a couple of chairs, and a bed. All cells are provided with barred windows. Contrary to the Brazilian institution, transgender women are allowed to wear female clothes all the time, use make-up, and to continue with hormonal treatments.

The prison itself is located in a slightly inhabited zone outside the little mountain town Belluno, in the Western Dolomites of the Italian Alps (Veneto region, northern Italy). All interviews were conducted inside a multi-purpose room of the transgender wing, normally used for lessons and other leisure activities, with a few desks and chairs, and a series of posters and geographical maps all along the partitions. The language used for the interviews was mainly Italian, though those conducted with the Brazilian inmates (three out of four) contained various Lusophonic inflections.

#### Naples-Poggioreale

This enormous prison complex in southern Italy is one of the most ancient (instituted in 1905) and largest (over 65,000 square meters) in the whole country. As underlined by the Committee on Civil Liberties, Justice and Home Affairs of the European Parliament (2014), this penitentiary context is a very complex institution. Today, it counts more than 2,000 inmates, distributed in eight sub-wings. The number of transgender people at the moment of data collection (July–September 2016) was seven, and all are detained in a protected section of the “Torino” sub-wing. The prison is located in a central, densely inhabited region of Naples, quite near the train station. As in Belluno, also in the parthenopaeus jail, all transgender inmates have individual cells with their own bathroom. Transgender women are usually allowed to freely circulate inside their section, without closing the doors of their cells. Like in Villa Baldenich, as in Poggioreale, they are allowed to wear female clothes, use make-up, and continue with their hormonal treatments.

The interviews were all conducted in the multi-purpose room of this small section. The room was quite empty with a few chairs and some equipment for fitness activities.

#### Florence-Sollicciano

The third Italian penitentiary complex is the prison (Nuovo Complesso penizenziario; NCP) of Florence-Sollicciano (central Italy). The NCP is situated in a recently constructed, densely populated suburb on the western hills of Tuscany's capital. Completed in 1982, according to a national reform of penitentiary legislation (Law 663), its architecture was inspired by the coat of arms (“giglio,” or lily) of Florence, as an explicit symbolic reference to the historical and cultural context, and moreover, to the institutional power. NCP's overall structure consists of a male and female wing, each one divided in several sub-wards and sections. During the data collection between February and April 2012, 1,021 inmates were detained in the institution, though the regular capacity was conceived for only 447 prisoners (Associazione Antigone, 2012).

The male part consists of a penal ward, a juridical one, and a clinical center, along with areas devoted to leisure, educational and physical activity. The juridical ward is split into eight sections, and the penal one into five. The sections of the penal and juridical wards contain ~18 large cells of about 12 m^2^, respectively, principally conceived for individual detainment but because of the prison's overpopulation they were occupied by three inmates.

The female wing is composed of three wards: two juridical and a penal one, along with, as for the male part, a series of zones used for sports and leisure activities. The protected sections, in addition, are devoted to the following categories of prisoners: women with psychiatric illnesses, mothers with children up to 3 years old, and transwomen. The original project conceived the protected section for transpeople inside the male section (like in all other Italian prisons), but recently it was moved to the female section.

#### Analytical procedures

As the corpus of data consists of the transcriptions of 23 in-depth, audiotaped interviews, a quali-quantitative analysis of the implicit and explicit contents, respectively, was performed throughout the methodological lens of a socio-cognitive approach devoted to a critical understanding of the production of discursive processes (van Dijk, 1993). Such a perspective allows an investigation into the symbolic means, the adoption of specific linguistic repertoires, the rhetoric strategies, and the argumentative logic underlying the production of a situated discursive event. Accordingly, the critical discourse analysis (CDA) considers language not just as a sophisticated communicative representation, due to specific cognitive functions, but rather as a powerful rhetorical (meta)artifact, strategically and intentionally adopted for transmitting intra-, inter-, and extra-personal means either to the *generalized* or the *significant* other.

Scholars such as van Dijk (2006) or Wodak (2007), among others, give special attention to the interdependence between cognitive functions, activated for the *intra-personal* processing of linguistic means and contextual elements, like norms, socio-economic background, ideological constraints, and cultural-historical coordinates, responsible for the *inter-* and *extra-personal* construction of discursive meanings. In that regard, the link between cognitive organization of linguistic skills, activated by the long-term memory (e.g., schemes, scripts, contexts) along with specific rhetorical strategies, have been considered for demonstrating how such connections could be responsible for the (re)production of stereotypes, prejudices, ideological orientations, etc. Different studies focused on the narratives of transgender people, already used computer-assisted programs for performing text-mining analysis, in order to bring forth statistically significant correlations between some occurrences and specific lexical classes (Speer and Parsons, 2006; Hochdorn et al., 2015, 2016). Such correlations, indeed, constitute a proper linguistic, semantic and semiotic genre, which allows to understand the discursive dimension of historically anchored and socially situated identities (Wodak, 2007).

As the current study endeavors to show how discourse builds up semantic meanings, which are socially shared and normatively legitimated, the intersectionality between gender, race, and class has been investigated to access the interdiscursive recurrences, throughout which transgender people detained in prison develop a negation of their selves. Such a negation could be expressed through the internalization of transphobia (Amodeo et al., 2015) on one hand, and when their skin color is black, xenophobia on the other (Reisner et al., 2014). The principal aim of the study, indeed, consists of demonstrating how the micro-linguistic properties of language promote discursive-based inequalities of power among socio-economic classes, cultural groups, gender identities, etc., because “racism, like sexism, is a very complex social phenomenon. There are fundamental social, cultural and economic dimensions to racism that have little to do with text or talk” (van Dijk, 2005: 3).

#### Investigation tool

The software package IRAMUTEQ, adopted for the current study, consists of an open source, multifunctional and statistic program for the quali-quantitative analysis of textual data (Ratinaud and Marchand, 2012). A detailed description of the software's functions is available elsewhere (Camargo and Justo, 2016).

Among the various analytical tools contained in the package, two functions have been used:

1. The hierarchical descending classification (HDC) of lexical classes (Reinert, 1990)
2. A word cloud analysis (WCA) of lexical frequency and distribution (Atenstaedt, 2017)
  1. The HDC consists of a descriptive statistical procedure, based on calculating the most frequent terms (occurrences) across the overall corpus of data and across specific lexical (stable) classes, individuated by the program ex-ante according the intertextual affinities of certain vocabularies, which are associated with an integrated dictionary (Italian and Portuguese in the case of the current study). This method reduces single textual segments into specific morphological key-terms, classified by the programs either regarding its relevance or its frequency.
  2. The WCA, in addition, aggregates the occurrences regarding specific lexical means by visualizing them graphically according to their frequency and intertextual relevance. It consists of a basic lexicographic analysis, while offering a well-structured output, particularly useful for the critical understanding of statistical procedures applied to qualitative, textual data.

#### Corpus of the textual data

The size of the two corpuses of data, N1 (16 Italian interviews) and N2 (7 Brazilian interviews), respectively, was determined by the sum of all absolute frequencies (occurrences) that constituted the lexical database. Regarding the pre-processed lexical corpus, the overall size counted 68,424 occurrences. From this pre-processed textual repertoire, all irrelevant lexical expressions were previously removed. Specifically, the following occurrences were not considered for the analytical procedures: demonstrative, indefinite, possessive, and additional adjectives; adverbs; articles; digits; conjunctions; onomatopoeia; pronouns; prepositions.

#### Data processing

The set of different occurrences (forms) defined the vocabulary contained in the corpus of the transcribed interviews. The number of identified forms V was 2,222 for the Brazilian (Portuguese language) sample and 5,136 for the Italian (Italian language) one, which was calculated by summing the number of different forms in the lexical corpus:

$$V=\sum_{i=1}^{Fmax}V_{i}$$

The lexical extension has been determined by type-token ratio (TTR) as a percentage ratio between the vocabulary size (V) and the size of the corpus (N). Such procedures show the lexical richness of the textual repertoire. The computed value for the analyzed text was 84.47% for the Italian data and 78.50 for the Brazilian one.

The hapax referred to those occurrences that appeared only once in the corpus; the percentage of hapax in the overall textual repertoire was 19.94 for the Italian sample and 20.88 for the Brazilian one.

The frequency class consisted of a set of words that showed the same number of occurrences throughout the overall corpus. For calculating the frequency classes, all words must be sorted according the number of occurrences in descending order. The most frequent word is associated with rank “one.” Moreover, the keywords refer to uncommon terms that appear with higher frequency across the overall corpus.

The algorithm that underlies the lexicometric analysis is based on a series of bi-partitions, due to a correspondence analysis conducted on a binary table (absence/presence) that crossed the textual units. Each bi-partition was carried out in three steps:

- A correspondence factor analysis (CFA) was conducted on the table. For all possible partitions along the first factor of the CFA, the inter-class inertia was calculated. An initial cutoff was performed on the partition for maximizing the inter-class inertia.
- Each unit of the table was initially switched from one class to another, and the inter-class inertia was recalculated afterwards. Whether the calculated values resulted in being higher than the previous inter-class inertia, the permutation has been retained. This part of the algorithm looped back around, until no permutation increased the inter-class inertia.

The principal aim of the algorithm was isolating those lexical forms that maximized inter-class inertia. In this context, the coordinates of lexical units on the first two axes were reported in relation to the percentage of cumulative variance explained by the mean of all four factors, namely 66.93 for the Italian sample and 68.68 for the Brazilian one.

## Results

The results section has been split into three subparagraphs, each one concerning one of the different analytical procedures that were performed.

In the first paragraph the findings of the HDC will be presented, while in the second one, the results of the WCA will be shown. Finally, a total of four significant extracts (one for each institute) were selected out of the interviews, to show how the two main key-terms (not and exist)—which emerged from the lexicographic analysis—appear within talk-in-interactions (Schegloff, 1998).

### Hierarchic descending classification (HDC)

**Figure 1:**
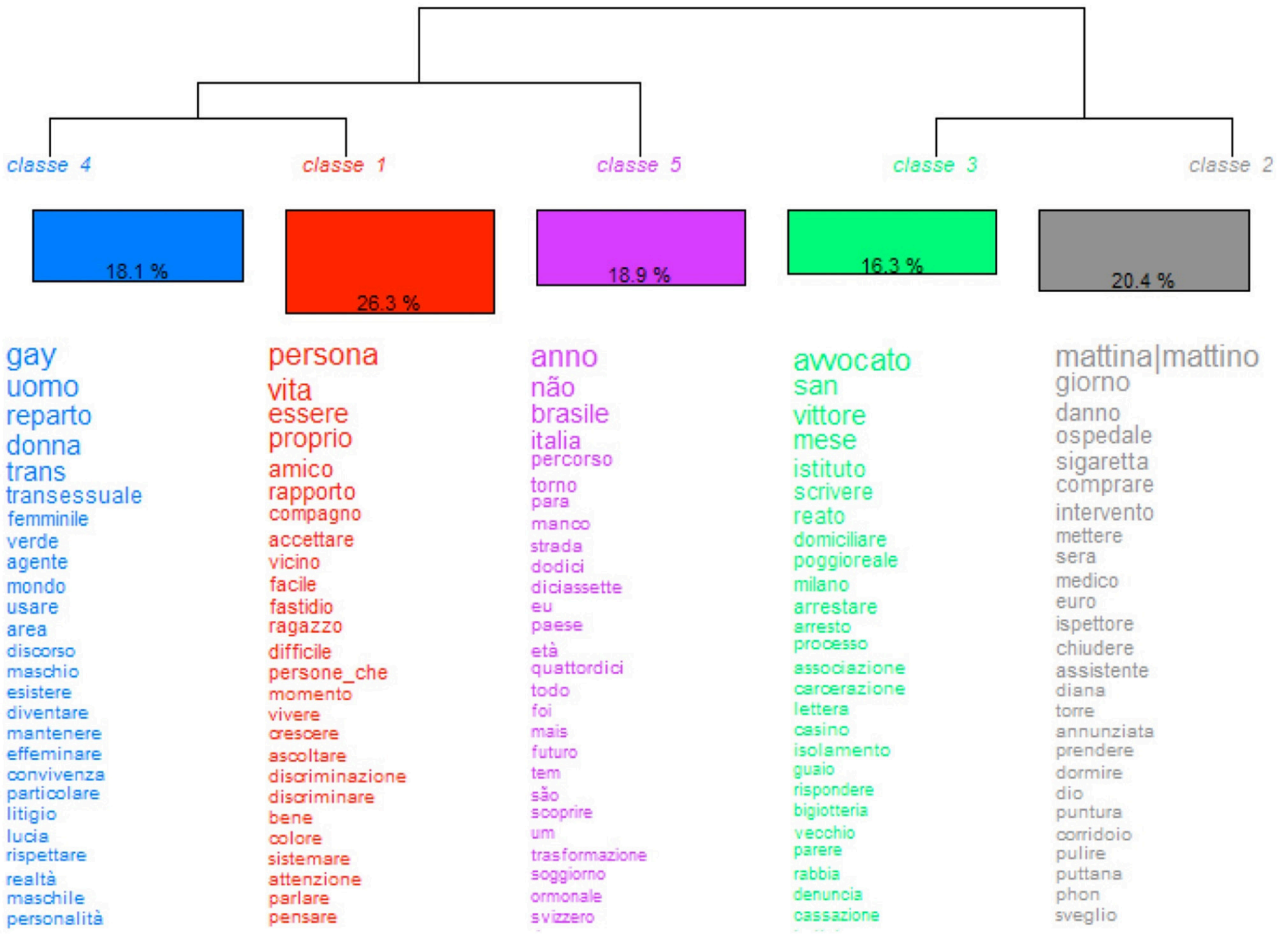
HCD of the Italian textual data.

The HDC individuated five stable classes for both national samples. Each of these classes contains the most frequent occurrences, related to a specific lexical content. The distribution and frequency of the overall key terms are presented in Figures 1, 3 for the Italian and Brazilian data, respectively.

#### HDC of the Italian lexical data

From the interaction between the five stable classes, two inter-class correlations emerged, namely, between classes four and one, on one hand, and between three and two, on the other. Class five correlates indirectly with the classes four and one, while the interdependence between these three classes interacts indirectly with the dyad of classes three and two.

The most representative class is class one with 26.3%, followed by class two with 20.4%. Class three, instead, shows the lowest averages with 16.3%, while classes four and five have nearly the same value (18.1 and 18.9%, respectively).

The most frequent occurrences contained in class one are: “persona – person,” “vita – life,” “essere – to be (exist),” “amico – friend,” “rapport – relationship,” “compagno–boyfriend,” “accettare – accept,” and “vicino – close.” Such words allude to an intimate and private representation of the self by emphasizing the primary status of a subjective personality, rather than that of a prisoner and/or a transgender, generally assigned by the common sense of a heteronormative-based, middle-class community. The desire to be considered and treated as a human being, and not just as an abnormal criminal, shows how transgender people must fight daily for their right to be recognized and for social inclusion. The affective value of interaction, instead, links to the most individual dimensions of one's existence, and surely, the most authentic representation of oneself and significant others. As class one is the most representative of the whole Italian sample, the words contained in this class certainly dominate the overall discursive structure. This shows that emotions, relations, and effects become the cardinal coordinates, circumscribing a person's central representation of oneself, consistently with the findings of former studies focused on this issue (Hochdorn et al., 2014).

Class two, being the second most representative, mainly contains occurrences linked to daily routines of one's life in transition inside the prison environment: “mattina/o – morning,” “giorno – day,” “ospedale – hospital,” “sigaretta – cigarette,” “intervento – intervention,” “medico – physician,” and the estrogenic hormone “diane.” Indeed, besides the intimate representations of one's private life (class one), what becomes one of the most crucial aspects of a transgender person's existence regards all treatments that are carried out to complete the sexual transition. Such treatments consist, indeed, in all those daily routines that a transgender woman must do for the continuity of her gender claiming, as clearly shown by words like “hospital,” “(surgical) intervention,” “physician,” and the estrogenic integrator “diane.” The substantive “cigarette,” finally, becomes an object of pleasure on one hand for attenuating the extreme stress factors of detention, while on the other, it is a pastime that prevents the idleness of prison life. In addition, a cigarette, such as other drugs, becomes an interactive and negotiation medium, often useful for empowering positive attachment styles with the other inmates. In general, drugs often become part of the living experiences of transgender people who have to face a transition between and across the gender antipodes, especially of those who belong to poor socio-economic classes, such as Black people (Reisner et al., 2014). The dealing of drugs, as shown by former studies, often becomes a further opportunity for gaining the money that are necessary for the highly expensive hormonal and surgical treatments on one hand, and because of the lack of professional opportunities on the other (Schilt and Westbrook, 2009). Especially Black transgender people, as shown by studies realized in Brazil (Hochdorn and Faleiros, 2017), suffer a further discrimination and exclusion when compared to transgender people belonging to higher social classes and other racial groups.

Indeed, many transgender people detained in prison—not only in Italy but also abroad -, associate their sex-working activities to drug-dealing. Such crime, indeed, mostly constitutes one of the main reason for their detainment (Reisner et al., 2014; Hochdorn et al., 2016).

The class with the third highest percentage is class 5, containing mostly words related to one's life experiences. Indeed, the most frequent lexical forms refer to nouns such as “Brasile – Brazil” and “Italia – Italy,” which, whether linked to the noun “percorso – journey,” underlie a life in transition not only between genders (man and woman) but also between countries (Brazil and Italy), because out of 16 transwomen interviewed in the Italian jails, nine were from Brazil and migrated to Italy, where some of them lived without a proper visa – “permesso di soggiorno.” Other words contained in this class, are “strada – street,” referring to street working activities (mostly prostitution and drug-dealing) and ages, like “diciasette – seventeen,” and “quattordici – fourteen,” which indicate the moment of becoming conscious about their own gender identity. Interesting is the use of the Portuguese inflection “não – no”, which is be the second most frequent occurrence inside this class. The “no,” indeed, becomes a global negation of oneself in many aspects of their lives.

Class 4, instead, contains mostly terms focused on their (trans)gender identity and sexual orientation, like the nouns “gay”, “uomo – man”, “donna – woman”, “trans”, “transessuale – transsexual”, the adjectives “femminile – feminine” and “maschile – masculine,” and the verbs “diventare – to become” and “effeminare – to feminize.” The words actually demonstrate the difficult challenge transgender people have to face when they try to claim their identity and sexual choices. The correlation between “gay” and “trans,” indeed, shows how common sense already associates gender identity with sexual orientation, consequently classifying transgender women as homosexual, cis-gender men.

The class with the lowest percentage is class 3, containing lexical forms linked to the daily practices in prison and to their general juridical situation. The most frequent words, indeed, are the nouns “avvocato – lawyer,” “instituto – institute,” “reato – crime,” “domiciliare – house arrest,” and “arrestare – to be arrested” and the names of important penitentiary institutions in Italy, like “San Vittore” in Milan or “Poggioreale” in Naples, where many transgender people have been detained before being transferred to other jails. Surprisingly, this last class, though containing words that are directly linked to the prison context where the all interviewed people must spend considerable time, results in being the least significant one. Such an apparent contradiction is due to the situational frame of a prison, which becomes for inmates an everyday context—where to interact and live. As shown in previous studies (Hochdorn et al., 2014), indeed, the contextual coordinates of a situated, interactive process become mere secondary aspects, whether compared to the private sphere or other important dimensions of one's life history.

#### HDC of the Portuguese (Brazilian norm) lexical data

**Figure 2:**
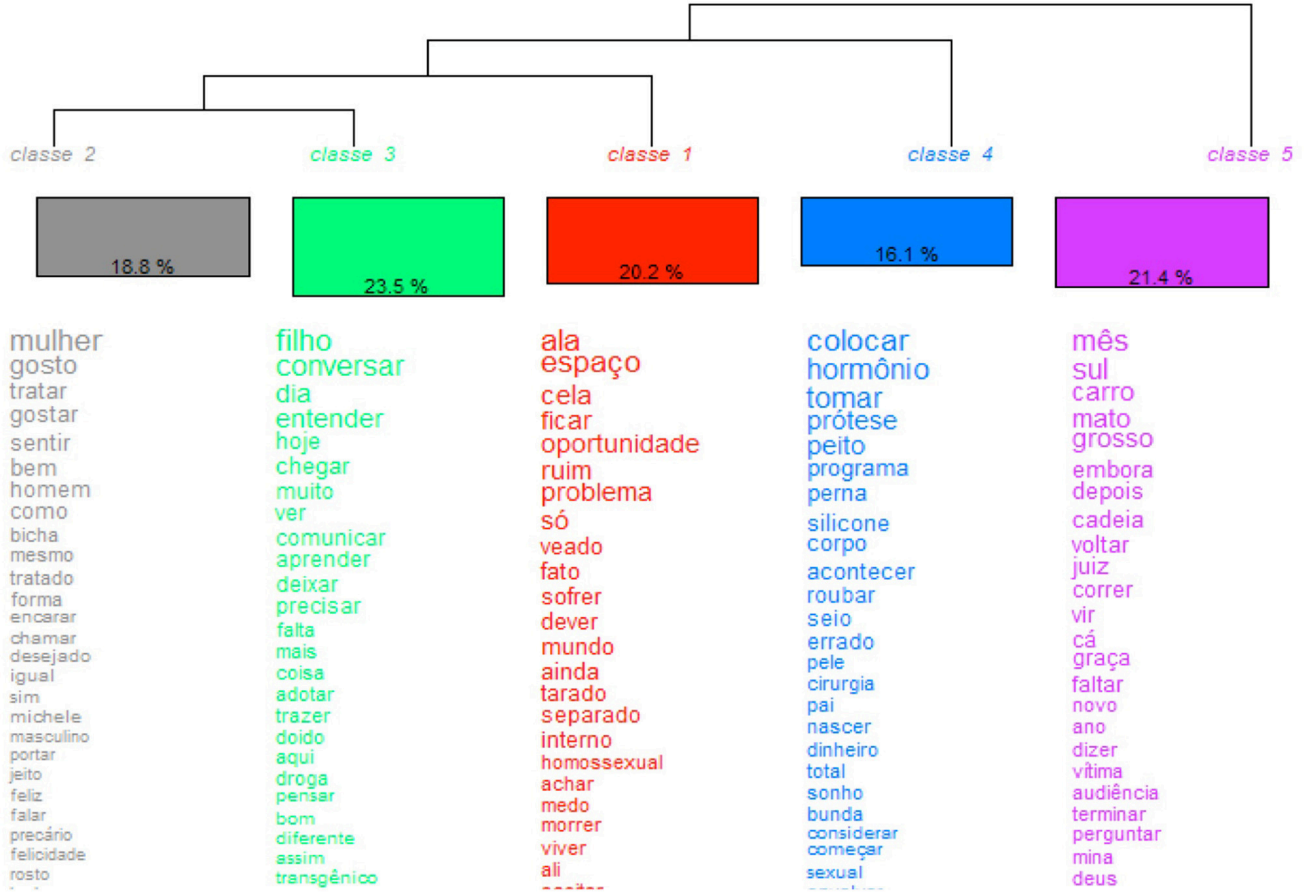
HCD of the Portuguese textual data.

The interaction among the five stable classes (Figure 2) shows a bilateral interdependence just between classes two and three, while all other classes only depend indirectly on each other. Class one is linked to the dyad of classes two and three and, furthermore, to class four, which is finally related to class five.

The most representative class is class three with 23.5%. Likewise the Italian sample, as for the Brazilian context, the class containing the highest number of occurrences is the one with key terms, alluding to intimate and private dimensions, as shown by the words “filho – son”, “converser – to talk”, “entender – to understand,” and “comunicar – to communicate,” among others. Contrary to the Italian sample, where none of the interviewed inmates were parents, in the Brazilian sample, four out of seven transgender people declared to have biological children, conceived with a cis-gender woman.

Class five (21.4%), contains words linked to daily habits before being arrested. This class mainly contains lexemes, referring to the conditions that contributed to committing the crime. Examples of such occurrences, are the nouns “Mato Grosso (a Brazilian federal state)” and “carro – car”, which refer to the way inmate 6 was arrested, namely the alteration of the car's registration number in this region. Accordingly, other terms contained in this class are “cadeia – jail,” “juiz – sentence,” and “audiência – judicial hearing,” which certainly refer to the antecedents, promoting their experience of detention.

Class 1 (20.2%) is the class containing those lexemes that refer to the contextual elements of the prison where the interviewed people are experiencing their period of detention. The most frequent terms, indeed, are “ala – wing,” “espaço – space,” and “cela – cell,” confirming that the restriction of one's physical agency has a considerable impact on their own representation of themselves, as also shown by previous studies (Hochdorn and Cottone, 2012). Keywords linked to feelings of rejection and inappropriateness enhance a situation of ulterior exclusion, in addition to the extremely difficult conditions of detention itself. Such lexemes, distributed as an interdiscursive Leitmotiv across the overall corpus of data are: “problema – problem”, “ruim – bad”, “veado – gay” (very offensive), “separado – separated,” which causes sorrow (“sofrer”) and fears (“medo”) of dying (“morrer”).

Class 4, furthermore, contains all occurrences linked to the transition between sex and gender. Such key terms, certainly, are “hormônio – hormone,” “prótese – protease,” “peito and seio – breasts,” “silicone” – same spelling as in English, “corpo – body,” “cirurgia – surgery” and “bunda – ass.” Interestingly, such words are related to “robar – to steal” for gaining “dinheiro – money”, in order to achieve their “sonho – dream” to be “nascer – (re)born” as a woman.

Occurrences contained in class 2, finally, allude to their desire (“desejado”) to be recognized as a woman and human being. Such words, indeed, are the noun “mulher – woman”, the verbs “gosto” and “gostar – I like, to like”, “igual – equal”, and “felicidade – happiness” in order to “sentir bem – feel fine”.

### Word cloud analysis (WCA)

The graphical output of this lexicometric procedure is based on a simplified factor analysis, according the most relevant occurrences across the overall corpus of data. Contrary to the HDC, based on an inter-class inertia, this kind of analysis brings forth the centrality of certain key terms. In the case of the current study, such words are “essere – to be/to exist” for the Italian sample and “não – no/not” for the Brazilian one.

#### WCH of the Italian lexical data

**Figure 3:**
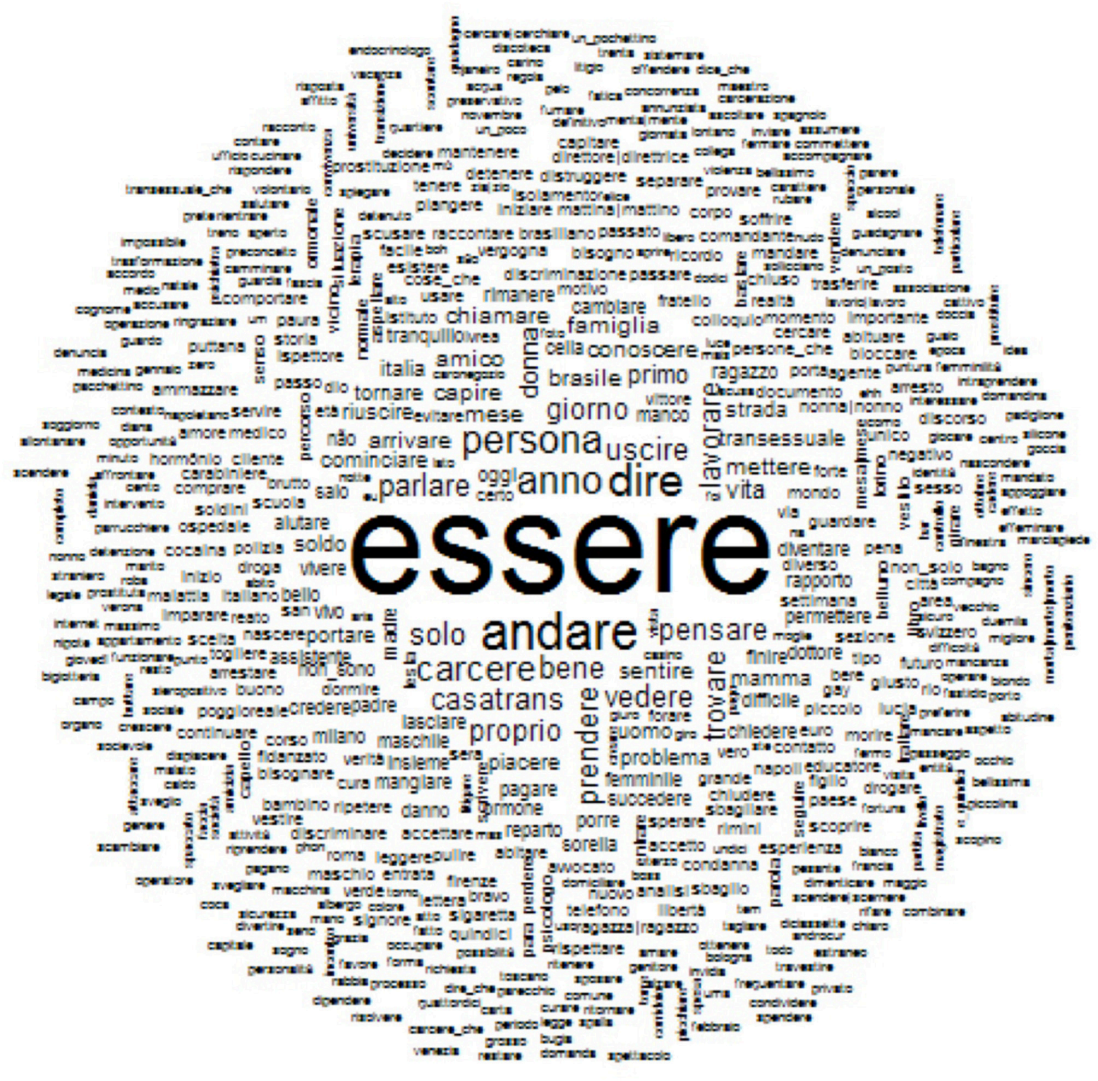
WCA of the Italian lexical data.

The central term of the Italian sample consists of the word “exists,” showing the relevance of such a semantic (and semiotic) construct of one's representation of self (see Figure 3).

Indeed, the most related and statistically relevant terms, surrounding such a central concept, are “persona – person,” “carcere – prison,” “anno – year,” “vita – life,” “diverso – different,” “diventare – to become,” and “pensare – to think.”

Such a lexical cloud surely shows, even in a situation with so much struggling, like the time (“anno – year”) spent in jail, a representation of their selves as a human being. They *exist* in prison as a person, and, consequently, not just a transgressor. They *are* different as a *person*, and not simply as an abnormal being; they are able to *think* and, therefore, to decide with their own, subjective agency, according to their choices about their life, e.g., to *become* – “diventare” – a woman.

The central concept of the Italian sample, accordingly, alludes to a perception of oneself linked to subjectivity and, consequently, agency.

#### WCH of the Brazilian (Portuguese) lexical data

**Figure 4:**
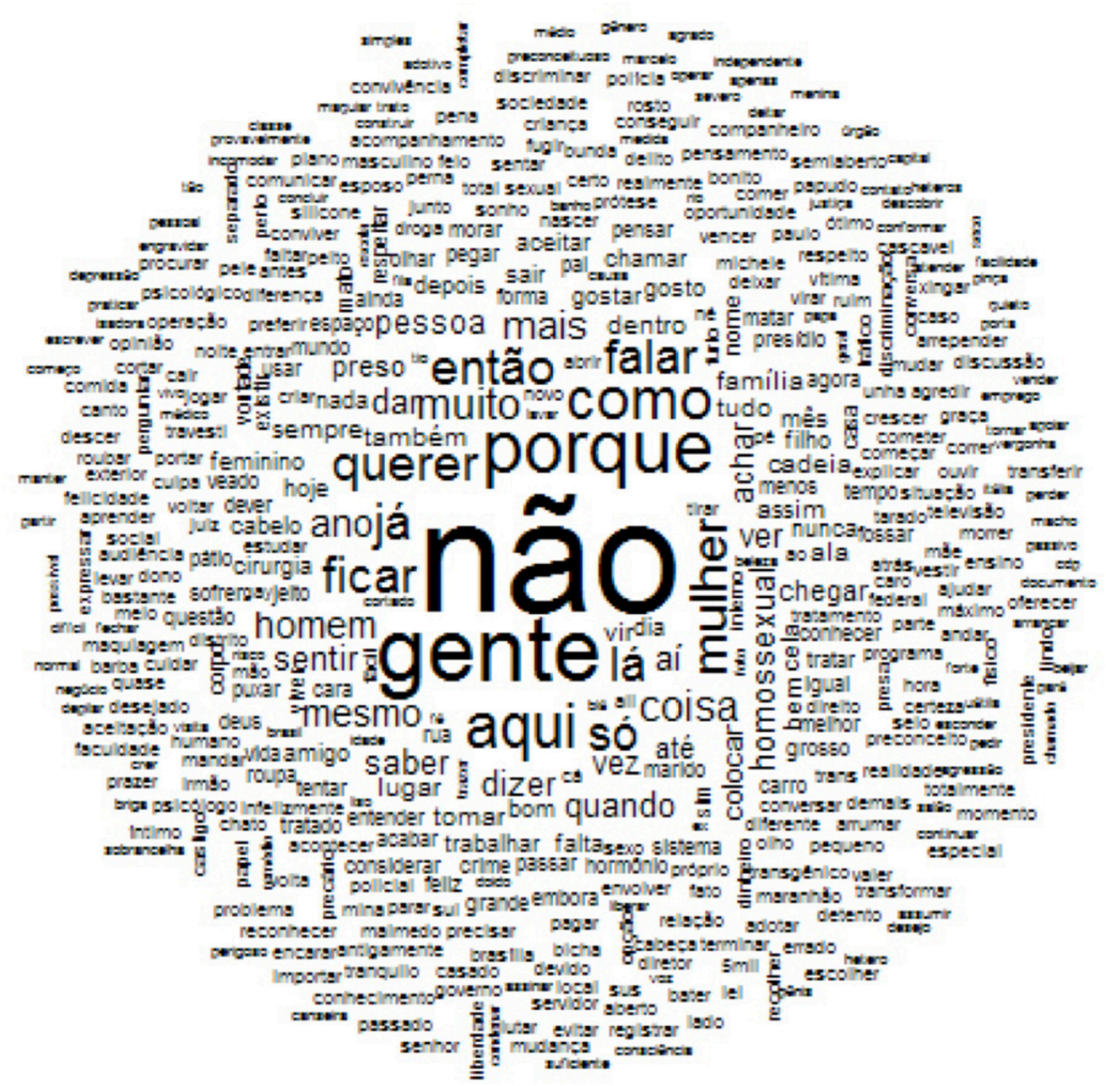
WCA of the Portuguese textual data.

Something quite different emerged from the Brazilian sample. The occurrence appearing most frequently across the overall corpus of data is the word “não – not/no” (see Figure 4). In a first analysis, it was found that the adverb “não” is often used in Portuguese as simply a connective term at the end of a sentence. Therefore, all such merely talk-in-interactions have been taken out before performing the WCH once again. Also, after this second analysis, however, the negation “não” continues to result as the most frequent occurrence. Indeed, the transgender inmates' representation of themselves is linked to a general feeling of hetero- and auto-rejection.

The related terms of the central occurrence, in addition, allude to continuous self-representations of negation, either inside or outside the prison walls. Transgender people, indeed, are not recognized either as women (“mulher”) on one hand nor as men (“homem”). Furthermore, they are even excluded from the LGB universe, because they do not match with social representations linked to homosexuals (“homossexual”). These last ones, though suffering several forms of symbolic and physical violence within the heteronormative domain, are more likely associated with culturally legitimated and historically anchored categories of social means, i.e., gay and lesbian people. Transgender women in the Brazilian prison, consequently, seem to denounce a multiple exclusion; they are banned in society as criminals, banned in culture as queers (“viado”), and banned in the LGB community as transgender. Such individuals, therefore, feel as if they are something different, which the generalized other does not even consider as people (“gente”). Finally, they have no voice in public life “não falar” (they don't talk) and, consequently they have no perspectives (“querer – to desire/wish”) for their private (“família”) or professional (“trabalho”) life.

### Analysis of talk-in-interactions

This last paragraph of the results section highlights, throughout, four of the most significant extracts (each for one prison context) associated with the lexicometric procedures of the Iramuteq software within small portions of textual content, how the two main key terms of the WCA (“não” and “essere”) interact within so-called contexts of situated *talk-in-interaction* (Schegloff, 1998).

#### Male prison of Belluno-Baldenich (Northern Italy)

The first extract, is from the interview with one Brazilian inmate out of the four inmates (three from Brazil and one from Italy), detained during data collection in the aforementioned prison. This talk-in-interaction refers to the description about her self-representation as a gendered agent.

Extract 1
Io vivo il mio **essere** molto bene. So quello che **sono**, so quello che voglio.
*I'm feeling fine with my way of* ***being****. I know who I* ***am****, I know what I want*.

In line with the results of the WCA, the representation of self across the Italian sample has been linked to the central concept “essere – to be.” The perception of self, accordingly, in this small and familiar prison context in the Dolomite Alps, is mostly positive. Like in the current extract, all inmates detained in this jail claim a egosintonic representation of their gender identity, which is not felt as something socially banned, but rather as an authentic expression of one's own way of being, thinking, feeling, and interacting. The interlocutor actually knows about her inner and deep aspirations concerning her life choices. Her transgender being, accordingly, shows a positive attachment style, either with herself, or with the significant and generalized other.

#### Female ward of the penitentiary context of florence-sollicciano (Central Italy)

The following extract is taken from an interview with one Brazilian inmate, out of a total of 18 (nearly all form Brazil and only one from Italy) inmates, detained during data collection in this prison. As in the previous case, the following extracts regard her representation of the self as a gendered agent.

Extract 2
Io non volevo assolutamente **essere** me.
*I didn't absolutely want to* ***be*** *myself*.

This short talk-in-interaction shows an opposed representation when compared to the former extract. The interlocutor, indeed, declared not feeling comfortable with her identity as a transgender woman. This denial of oneself, certainly, became a redline of nearly all transgender inmates interviewed in the prison of Florence. Within this particular jail, transgender women are detained in a special section inside the female wing of the prison. Contrary to the initial hypothesis, which pretended to empower transpeople's agency in claiming a female-driven representation of the self within a feminine context, these inmates suffered more violence inside this section, if compared to those transgender women who are detained in special sections inside ordinary male prisons. As shown by previous studies (Hochdorn et al., 2016), cis-gender female inmates consider transpeople as a threat and, consequently, they do not recognize the gender claiming a female identity of the transwoman. Such lack of social concern from the other cis-gender female inmates makes transpeople less aware of their most authentic way of being.

#### Male prison of Naples-Poggioreale (South Italy)

The last extract from the Italian sample was taken from an interview with one Italian inmate out of the seven inmates (five from Italy, one from Brazil, and one from Algeria), detained during data collection in this prison. The following talk-in-interaction, refers to the description about her self-representation as a gendered agent.

Extract 3
Ma non è corretto che io possa fare la doccia (…) con le donne, perché non **sono** completamente donna; offende pure il mio **essere** donna.
*It would not be correct, however, to take a shower together with women, because I* ***am*** *not completely a woman; this even violates my own* ***being*** *a woman*.

Such an apparent contradiction also emerged in recent studies about transgender women detained in prison (Hochdorn et al., 2016). As referred to by several inmates who were interviewed for the current research, they do not completely feel as a woman, deconstructing, thereby, the binary vision of the gender antipodes. Transsexual people, indeed, are commonly seen as agents, who were born in the “wrong” body, and therefore, they desire to transition toward the opposite site of the sexual dichotomy. Such antinomy, however, could not be superimposable for all transgender people who do not necessarily consider themselves as completely associable to a social representation of a birth-assigned woman. The interlocutor of the present talk-in-interaction, as all other inmates who took part in this investigation, has not completed a sexual reassignment surgery. Therefore, as has emerged from the textual data gathered in the female prison context of Florence, the choice to co-locate women affirmed as transgender inmates in female wards, would not consequently grant the recognition of one's identity. The interviewee, indeed, stated, that a detention in common with cis-gender woman, might not match with her own transgender identity.

#### Male prison of brasília-Papuda (Brazilian Central Highlands)

The detention in a common penitentiary system, where transgender inmates, contrary to the Italian system, are detained together with male prisoners, evidently becomes the hardest of all imprisonment experiences, observed within the current study. As emerged from the WCA, the negation of self, expressed throughout the adverb “não” becomes the *Leitmotiv* of an harsh situation of avoidance and hiding. The following extract, taken from the interview of one of the seven inmates, detained during data collection in the Papuda prison, clearly shows how transgender people in Brazilian prisons create a discursive genre, linked to a complete denial of oneself. Such “auto-castration” of one's identity, indeed, does not only regard one's gender claim, but rather a complex and wired stigma that black people in Brazil have to face—namely, to belong to a socio-economic status, commonly associated with crime, anti-social behavior, violence, and poverty.

Extract 4
Já fiquei com presos comuns, só que escondido. Eles **não** sabiam da minha opção. Em diferenças situações a gente tem que se portar de tal forma que eu **não** me sentia bem; até mesmo no pátio em dia assim de detentos que **não** gostam da gente. Eu **não** posso ficar da forma que eu gosto, dando pinta que eu sou bicha e tal.
*I have already been detained together with common inmates, but in a hidden manner. They did* ***not*** *know about my choice (to be a woman). In different situations, we must behave ourselves in a manner, which does* ***not*** *make me feel fine; even during daily small-talk, the other (masculine) inmates do* ***not*** *like us. I can****not*** *stay in the way I like to be, making the others believe that I am a perverse person or something similar*.

The adverb “não” within this short extract has been repeated four times, underlining the continuous struggle among one's right to undo gender in prison and the duty to adapt oneself to the constraints of a strongly hetero-normalized context. As the interlocutor, and all other transgender inmates of the Papuda prison stressed, being a transgender person in jail becomes a hidden and even clandestine condition. Neither the other inmates nor the surveilling agents, indeed, have any idea about what it means to face a transition among gender identities. Consequently, to avoid violence, excessive discrimination, and transphobia, these inmates must behave themselves as much as possible, in line with an inter- and extra-personal representation of a hetero-normalized masculinity. As underlined by Amodeo et al. (2015), such negative attachment styles are often interiorized by transgender people themselves, making masculine-driven behaviors become an intra-personal representation of a pathologic self (“bicha”), which must be normalized in socially, more legitimated manners. Accordingly, transgender women in Brazilian male jails are forced to redo gender, namely to act on the stages of daily prison life in the role of a masculinized identity, as it was originally assigned at birth. Such a negation of self, causes two kinds of reactions in these inmates:

1. Refusal of oneself, by adopting an etio-pathogenetic explanation: “I am wrong.”
2. Representation of oneself as a cis-gender gay man: “My sexuality is different, but my sex is right.”

## Discussion

In the current study the discursive processes of transgender people detained in Italian and Brazilian have been critically analyzed. The main goal of the study, accordingly, was to show how a (trans)gendered identity depends on the intersection among different social, cultural, relational, and normative dimensions. From the analysis, indeed, two main discursive positions emerged:

- A negation of self for the Brazilian sample, expressed through the Portuguese occurrence of “não.”
- An affirmation of self for the Italian sample, expressed through the Italian occurrence of “essere.”

The situation observed in Brazil, actually, reflects the general condition of detainment of transgender people in other countries, especially the USA (Samons, 2001; Jenness, 2010; Jenness and Fenstermaker, 2014), where self-harm (Brown, 2010), loss of self (Disspain et al., 2015), and extreme physical and psychological violence, received either from heteronormative and cis-gender male prisoners (Gruberg, 2014) or prison workers (Kirkup, 2009), are the daily rule. Such a situation as has been underlined by several scholars (Sexton et al., 2010; Jenness and Fenstermaker, 2014), is due to common imprisonment with heterosexual cis-gender men, like those transgender inmates who has been interviewed in the Brazilian jail for the current study.

By comparing different practices of detainment (separated sector in male prisons, separated sector of a female prison ward, common detention with cis-gender men) in two different countries, it was possible to shed light on how the identity of a person changes either according the situation of a certain context or according to wider systems of symbolic means and normative power.

In particular, Brazilian transgender people in Italian prisons show a more authentic and egosintonic representation of their gendered self, while those detained in Brazilian jails, though the cultural origin and the skin color (black) is the same, often negate their feeling as a transwoman, and instead, represent themselves as cis-gender, homosexual men. Something similar has also been observed in many US prisons (Sultan, 2003; Brown and McDuffie, 2009; Alexander and Meshelemiah, 2010; von Dresner et al., 2013; Jenness and Fenstermaker, 2014), confirming that transgender experiences (as an identity claiming) are still considered as superimposable to homosexuality (as a sexual orientation or preference; Hochdorn et al., 2016). Brazilian inmates in Italian jails, indeed, had to reach Europe, and, must, therefore, face a migration experience. Such people, when compared to those detained in Brazil, already had access to formal education and, in some cases, regular working experiences. A higher educational level, indeed, promotes higher levels of self-consciousness on one hand, while on the other, they could attend special courses in prison (like university courses), could be engaged in more complex working activities (despite cleaning), and have better chances for social reassignment. Transgender inmates in Italy, consequently, have projects for their future life, and, are definitely considering changing their previous living habits, especially dealing and consuming illegal drugs (Chianura et al., 2010; Hochdorn et al., 2016).

As most Brazilian transgender people, mainly those detained in the prisons of Belluno and Florence, migrated to Italy (and generally to other EU countries) to improve their own living conditions, their ambition of self-realization was, therefore, much higher than those incarcerated in the Papuda prison.

Generally, the situation in the Brazilian prison is much more dramatic and precarious when compared to the Italian institutions. In Italian prisons, although transgender inmates are detained in protected sectors, apart from the common, mostly male and heteronormative prison population, the incarceration process itself is quite different. In Italy, indeed, several reforms, above all from the 1980s onwards (Chianura et al., 2010; Hochdorn et al., 2016) have contributed to humanize the penitentiary system. Just a couple of structural differences among the prison administration of both countries could be pointed out (Tables 3, 4):

**Table 3:** General differences between Italian and Brazilian jails.

**Italian jails**	**Brazilian jail**
Individual cells, like in Belluno or Naples, or addressed for a maximum three prisoners, such as in Florence.	Collective cells for more than 20 prisoners.
Each cell is provided with a restroom and with barred windows, which allow a restricted view outside.	The water closet is in the same place as where people are sleeping and the cells have no windows for looking outside.
Prisoners do not have to wear uniforms or cut their hair.	Prisoners must wear uniforms (white for common inmates and orange during working activities) and a haircut is obligatory.

**Table 4:** Differences regarding the detention of transwomen between Italian and Brazilian jails.

**Italian jails**	**Brazilian jail**
Transwomen are detained in protected sectors.	Transwomen are detained in common sectors with cis-gender men.
Transwomen are allowed to wear female clothes, use make-up and maintain their long hair.	As for cis-gender male prisoners, transwomen must wear male uniforms and cut their hair. They cannot use make-up.
Transwomen can continue with their hormonal treatments inside the institution. Such treatments are partially supported by local health policies.	Transwomen cannot continue with hormonal treatments, even if they had regularly taken them before getting arrested.

There are some other general aspects, instead, that are in common among the two penitentiary systems:

- Most prisoners, either in Italy or Brazil, have a dark skin color and normally belong to low income or extremely precarious socio-economic backgrounds.
- Many prisoners faced a migration process; in Italy, they came from other countries, while in Brasília, they came from the poorer regions north and northeast of Brazil.
- The overpopulation of the inmates in nearly all institutions of both countries (except Belluno) results in twice the regular capacity of the institutions (especially Florence and Brasília).
- Self-harm and suicide are common aspects in nearly all jails of both countries.

Finally, also regarding the specific detainment condition of transwomen, some recurrences could be found out for both countries:

- Most transgender prisoners, not only in Brazil, but also in the whole Italian penitentiary system, are from South America; in the specific case of the current study, out of 16 interviewed inmates, nine were from Brazil, one from Algeria, and only six were from Italy (nearly all from Naples and other southern regions of the country).
- All Brazilian transgender inmates, as most of the general prison population (see above), in both countries are black.
- Nearly all transwomen of both countries referred to rejection and refusal of their gender choice in their family context.
- All transwomen in both countries had sex-work experiences.
- All transwomen in both countries suffered extreme psychological, social as well as physical violence.
- The most common crimes committed by transwomen in both countries are drug-dealing and sex-work exploitation.

The situation observed in both countries, accordingly, showed the emergence of two different ways to face the detention of transgender people in prison. In Italy, where since 1980 special attention has been paid to the incarceration of transpeople, such inmates live their detention in a more harmonious manner compared with the Brazilian sample.

Furthermore, as mentioned by some Brazilian prisoners, a last variable problematizes even more their life in jail: being black. The skin color in those countries, which has an economic system based on the exploitation of black people exported from Africa (slavery), such as Brazil or the USA, nowadays is a delicate, struggling, and complex challenge to be faced. The social and cultural discrimination of black people in the USA and in Brazil (among other countries worldwide), causes a vicious cycle among exclusion and denial, promoting a ghettoization of black people within social, cultural, and economic poverty. Such a condition, as has been shown by various studies conducted in both countries (Reisner et al., 2014; da Silva and Oliveira Lima, 2016; Wildeman and Wang, 2017), reifies a strict and dangerous link between crime and race.

In Italy, however, above all, since protected sectors for transgender inmates were instituted, the detainment experience is less traumatic, dramatic, and sharp compared to the Brazilian reality. As has been pointed out by few previous studies about this reality in Italy (Chianura et al., 2010; Hochdorn et al., 2015), the protected sectors improved the situation either of the transgender prisoners, who suffered less violence and discrimination, or of the prison workers, who received special courses for interacting in an appropriate manner with these inmates. Regarding the skin color, the Brazilian inmates in the Italian jails, compared to the white-Caucasian Italian prisoners, discussed an explicit discrimination in their own country of origin, while racial discrimination in Italy is perceived in a more silent and implicit way. The main findings of the lexicometric analysis clearly show how such a discrepancy among a rejection of self, like in the Brazilian prison, and a more egosyntonic way to manage an auto- and hetero-acceptance of a (trans)gender identity in the Italian jails, promote different intra-, inter-, and extra-personal representations of one's own sexual identification. The two cardinal discursive positions that emerged from the data-analysis, namely “not” and “exist,” could therefore be considered as a quasi *Hamletian* existential dissonance either on a cognitive-psychological level or on a socio-cultural one.

The different findings emerged from the current study, underlie the urgent need to grant for a substantial psychological, social, clinical and educational support for transgender inmates, detained in prison. As recent studies in clinical psychology showed (Amodeo et al., 2015; Vitelli, 2015), transgender people are particularly exposed to vulnerability, marginalization and a general loss of self. Such a viscous circle becomes even more evident, when these people must face a period of detention. Psychological counseling, for sure, must be implemented within the daily practices of prison life, in order to improve not only the detainment-condition of transgender people, but also the professional routines of prison-workers, interacting with these inmates. As shown by former surveys conducted in Italy (Chianura et al., 2010; Hochdorn et al., 2015), offering specific training programs, on a psychological and educational level, for prison guards and penitentiary staff more generally for making these professionals more aware about the complex existential situation of being a transgender person, granted for better interaction, lower levels of personal stress and more positive attitudes toward the self. Furthermore, also the working conditions of prison staff-members gained efficient and enduring benefits as well as lower levels of burn-out, after receiving a specific training support.

### Study limitations

The current study has several limitations. First, the samples of both countries are asymmetric: three prisons in Italy, only one in Brazil. There are two important reasons for the choice of such an uneven distribution. (1) In Italy there are different practices of detention for transgender inmates: separated sectors in small male prisons—so-called “low threshold jails (“carceri a bassa soglia”)”—like the prison of Belluno; separated sectors in huge male penitentiary complexes, like in Naples; and finally, separated sectors in female prison contexts, such as in Florence.

Because in Brazil, transgender inmates are co-located in common jails with cis-gender heterosexual men, it was not necessary to compare different institutions with different forms of detention, such as in the Italian jails. (2) In Brazil, transgender inmates are classified as men; therefore, it is quite difficult to get an approximate overview of how many transwomen are incarcerated and in which institutions they are detained. In Brazil, there is also a complete lack of scientific literature that focuses on this reality. To the best of our knowledge, no study, before the current one, has ever been published about transgender women in Brazilian jails.

Another limitation regards the choice of the investigation instrument. As the main goal of the current study consisted of a lexicometric analysis of the textual data (the interviews with the transgender inmates), this survey has principally focused on the discursive dimension of interaction on a syntactic and semantic level. The limitation of a quali-quantitative investigation tool, indeed, does not allow a deeper analysis on a semiotic, symbolic level. Future research should, therefore, conduct a critical analysis of the discursive processes for catching the implicit means “between the lines.”

Finally, due to the intercultural, comparative aims of the present survey, the two national specific samples were interviewed by using two different languages: Italian and Portuguese. The overall textual material, therefore, was split into two archieves, which must be processed separately in the Iramuteq software. Such a methodological limit allowed for only the interdiscursivity, but not the intradiscursivity to emerge, from the overall corpus of textual data.

### Final remarks

The current study highlighted the discursive processes, which underlie the narrative construction of self of transgender women detained in Italian and Brazilian jails. This survey is the first interdisciplinary and international research project about the incarceration of transgender inmates in both countries. What principally emerged out of the findings was a radically different discursive positioning between the two national samples. While the world cloud analysis for the Italian data showed that the most frequent occurrence was the verb “essere – to be/exist,” the Brazilian data showed the adverb “não – no/not” to represent the most significant term within the discursive production of the inmates in the Papuda prison. There are two main reasons for such a discrepancy in the discursive representation of self: transpeople in Italian jails are classified as such; therefore, it is possible to get an overview of how many transgender inmates exist in the Italian penitentiary system. Furthermore, transwomen in Italian jails are detained in protected sectors, and prison workers receive a special training to interact appropriately with these inmates. Transgender people in Italian jails, accordingly, are visible; indeed, they exist (“essere”). However, in Brazil, transwomen are detained together with cis-gender heterosexual men and, moreover, they are not recognized for their own authentic gender claim, but, instead, they are classified as men. Their identity in prison is, therefore, negated; their self becomes invisible and, consequently, they do not (“não”) exist.

Transgender people represent a complex challenge for prison administration, because it is not clear in which penitentiary context they might receive more recognition. Certainly, transgender women should not be detained in ordinary sections together with cis-gender men, like in Brazil, and feel free in (un)doing gender in jail, such as in Italy.

Finally, transgender people in prison should receive special attentions in order to face their special needs, which are radically different, whether compared to other typologies of inmates. Until adequate, multi-professional support to raise collective and individual awareness, either inside or outside the prison-walls, of the complex condition of transgender inmates is implemented throughout the healthcare system and clinical policies, neither effective nor enduring benefits could be expected from intervention programs to prevent (trans)gender based violence in jail. Especially within penitentiary contexts, psychological counseling with transgender women should pay a special attention to the several psycho-social dimensions of such a harsh and extreme existential condition. Such a paradigmatic and at the same time pragmatic view made emerge its inner complex articulation within different social, cultural and normative contexts.

## Author contributions

The all authors AH, VF, PV, and RV contributed to the drafting phases of substantial contributions to the conception or design of the work and interpretation of data for the work; drafting the work or revising it critically for important intellectual content; final approval of the version to be published; agreement to be accountable for all aspects of the work in ensuring that questions related to the accuracy or integrity of any part of the work are appropriately investigated and resolved. All data were personally collected and transcribed verbatim by AH in both countries; only in the prison of Naples the interviews were conducted together with an Italian transgender activist, who also transcribed them verbatim.

### Conflict of interest statement

The authors declare that the research was conducted in the absence of any commercial or financial relationships that could be construed as a potential conflict of interest.
